# Sleep, Fainting, Apoplexy

**Published:** 1870-09

**Authors:** 


### SLEEP, FAINTING, APOPLEXY.

When a man is asleep, his pulse beats and his lungs play, but he is without sense, and you can easily wake him up.

If a person "faints," he too is without sense, but he has no pulse and does not breathe.

Apoplexy is between the two; the heart beats, the lungs play as in sleep, and there is no sense, as in fainting, but you can't shake the man back to life.

In sleep, the face is natural.

In a fainting fit, it has the pallor of death.

In apoplexy, it is swollen, turgid, and fairly livid.

If a man is asleep let him alone, nature will wake him up as soon as he has got sleep enough.

When a person faints, all that is needed is to lay him down flat on the floor and he will "come to" in double quick time. He fainted because the heart missed a beat, failed for an instant, failed for only once to send the proper amount of blood to the brain. If you place the patient in a horizontal position, lay him on his back, it does not require much force of the heart to send the blood on a level to the head; but if you set a man up, the blood has to be shot upwards to the head, and this requires much more force; yet, in nine cases out of ten, if a person faints and falls to the floor, the first thing done is to run to him and set him up, or place him on a chair.

In apoplexy, as there is too much blood in the head, every one can see that the best position is to set a man up, and the blood naturally tends downwards, as much so as water will come out of a bottle when turned upside down, if the cork is out.

If, then, a man is merely asleep, let him alone, for the face is natural.

If a man has fainted, lay him flat on his back, for his face is deadly pale.

If a man is apoplectic, set him in a chair, because the face is turgid, swollen, livid, with its excess of blood.

